# The Gene Toolkit Implicated in Functional Sex in Sparidae Hermaphrodites: Inferences From Comparative Transcriptomics

**DOI:** 10.3389/fgene.2018.00749

**Published:** 2019-01-18

**Authors:** Alexandros Tsakogiannis, Tereza Manousaki, Jacques Lagnel, Nikolaos Papanikolaou, Nikos Papandroulakis, Constantinos C. Mylonas, Costas S. Tsigenopoulos

**Affiliations:** ^1^Hellenic Centre for Marine Research, Institute of Marine Biology, Biotechnology and Aquaculture, Heraklion, Greece; ^2^Department of Biology, University of Crete, Heraklion, Greece; ^3^Department of Medicine, University of Crete, Heraklion, Greece

**Keywords:** sex-biased gene expression, hermaphroditism, RNA-seq, comparative transcriptomics, gonads, brain

## Abstract

Sex-biased gene expression is the mode through which sex dimorphism arises from a nearly identical genome, especially in organisms without genetic sex determination. Teleost fishes show great variations in the way the sex phenotype forms. Among them, Sparidae, that might be considered as a model family displays a remarkable diversity of reproductive modes. In this study, we sequenced and analyzed the sex-biased transcriptome in gonads and brain (the tissues with the most profound role in sexual development and reproduction) of two sparids with different reproductive modes: the gonochoristic common dentex, *Dentex dentex*, and the protandrous hermaphrodite gilthead seabream, *Sparus aurata*. Through comparative analysis with other protogynous and rudimentary protandrous sparid transcriptomes already available, we put forward common male and female-specific genes and pathways that are probably implicated in sex-maintenance in this fish family. Our results contribute to the understanding of the complex processes behind the establishment of the functional sex, especially in hermaphrodite species and set the groundwork for future experiments by providing a gene toolkit that can improve efforts to control phenotypic sex in finfish in the ever-increasingly important field of aquaculture.

## Introduction

Sexual dimorphism is widespread in nature and describes the complete set of differences between sexes (Jazin and Cahill, 2010; Angelopoulou et al., 2012; Gamble and Zarkower, 2012). Despite differences in morphology and behavior, females and males are genetically nearly identical. However, many genes are more actively transcribed in one sex than the other, and sex-biased expression is considered to be the way by which sexual dimorphisms can arise from the genome (Ranz et al., 2003; Ellegren and Parsch, 2007; Mank et al., 2008; Parsch and Ellegren, 2013). The establishment of sexual phenotype is accomplished through the fine-tuned action and collaboration of numerous genes whose expression is prompted or repressed at preset crucial time periods during development (Angelopoulou et al., 2012).

Teleost fishes show great variations in the way the sex phenotype forms. Sexual differentiation in this group of vertebrates is evolutionarily flexible, as different sexual patterns exist, ranging from gonochorism to hermaphroditism. Studies in fish have already redefined our understanding of the complexity and plasticity of the sex determination (SD) process, yet detailed genetic information on SD genes is currently available for only a limited number of species (e.g., *DmY/Dmrt1bY* in *Oryzias latipes* and in *O. curvinotus* (Matsuda et al., 2002), *Gsdf* in *O. luzonensis* (Myosho et al., 2012), *Amhy* in *Odontesthes hatcheri* (Hattori et al., 2012), *Amhr2* in *Takifugu rubripes, T. pardalis*, and *T. poecilonotus* (Kamiya et al., 2012), and *SdY* in *Oncorhynchus mykiss* (Yano et al., 2012). Furthermore, sex-linked markers and/or sex-determining regions have already been reported in several species, including some that are of interest to the aquaculture industry, such as the catfish *Clarias gariepinus* (Kovács et al., 2000), the Nile tilapia *Oreochromis niloticus* (Ezaz et al., 2004), the rainbow trout *Oncorhynchus mykiss* (Felip et al., 2005), the turbot *Scophthalmus maximus* (Martínez et al., 2009), the half-smooth tongue sole *Cynoglossus semilaevis* (Shao et al., 2010), some salmonids (Iturra et al., 2001; Phillips et al., 2013; Larson et al., 2016), Gasterosteidae (Ross et al., 2009), species of the genus Eigenmannia (Henning et al., 2011), and the European sea bass *Dicentrarchus labrax* (Palaiokostas et al., 2015).

Sex control constitutes one important and favorably targeted area of research in aquaculture, as it has been one of the most important of the facilitators for large-scale industrial production (Budd et al., 2015). A large body of research has targeted sexual development in commercially important fish species in order to understand and control sex and be able to regulate sexual differentiation and maturation (Budd et al., 2015). Among aquacultured species, the Sparidae present a morphologically and ecologically highly diverse family of Perciform fishes. An exceptional characteristic of the species in this family is that they exhibit nearly every reproductive motif known in fishes, rendering them ideal for comparative studies. In addition to gonochoristic species (e.g., *Dentex dentex*), in which individuals retain the same sex through their lifetime, various forms of hermaphroditism have been recorded (Buxton and Garratt, 1990). Some species change from functional females to functional males (protogyny) (e.g., *Pagrus pagrus* and *Pagellus erythrinus*), while others function first as males and then change to females (protandry) (e.g., *Sparus aurata*). Furthermore, some species exhibit rudimentary hermaphroditism (e.g., *Diplodus puntazzo*), so-called “late gonochorism” (Buxton and Garratt, 1990), as individuals possess both male and female gonadal tissue during sex differentiation and before puberty, and one of the two gonadal types develops fully only after sexual maturation, with no evidence of further sex reversal.

With the advent of next-generation sequencing technologies, such as RNA-Seq (Wang et al., 2009), whole-transcriptome approaches have been used to study the molecular mechanisms of sexual differentiation in fish (Zhang et al., 2011; Forconi et al., 2013; Sun et al., 2013; Tao et al., 2013; Böhne et al., 2014; Liu et al., 2015; Casas et al., 2016). Regarding sparids, most studies on sex determination/differentiation and sex change have been conducted in protandrous species, such as the black porgy *Acanthopagrus schlegelii* (He et al., 2003; Wu and Chang, 2013) and gilthead seabream *Sparus aurata* (Loukovitis et al., 2012). The main focus of these studies is to track differences in gene expression between males and females and discuss the expression patterns of important genes in the general context of sex determination/differentiation and sex-change. Recently we sequenced and analyzed the brain and gonad transcriptome of the rudimentary hermaphrodite sharpsnout seabream *Diplodus puntazzo* (Manousaki et al., 2014) and of two protogynous sparids, the red porgy *Pagrus pagrus* and the common pandora *Pagellus erythrinus* (Tsakogiannis et al., 2018). In this study, we sequenced and analyzed the sex-biased transcriptome of two sparids with different reproductive modes: the gonochoristic common dentex *Dentex dentex* and the protandrous gilthead seabream *Sparus aurata*. Then, we explored the sex-specific expression patterns in all five transcriptomes from these five sparid species exhibiting four different reproductive modes. Using RNA sequencing, we analyzed and compared differences between male and female transcriptomes in gonads and brain, the tissues with the most profound role in sexual development and reproduction (Vilain and McCabe, 1998; Wilson and Davies, 2007) to obtain a global view of sex-biased expression in these tissues, and through comparative analysis to reveal common male and female-specific genes and pathways that are possibly involved in the development of the functional sex phenotype. Our results contribute to the understanding of the complex processes of sex differentiation, especially on hermaphrodite species.

## Materials and Methods

### Ethics Statement

Animal welfare was achieved according to the “Guidelines for the treatment of animals in behavioral research and teaching” (Anonymous, 1997) (see also Tsakogiannis et al., 2018). All fish utilized in the study were kept in registered and authorized facilities to maintain and perform animal experiments; rearing and sampling followed the guidelines of the Directive 2010/63/EU for the protection of animals used for experimental and other scientific purposes (Official Journal L276/33) (EU, 2010). In addition, experimental sampling protocols were approved by the IMBBC's aquaculture department committee and methods were in accordance with relevant guidelines and regulations approved by the Hellenic Ministry of Rural Development and Food and the Regional Directorate of Veterinary Medicine for certified experimental installations (EL 91-BIO-04) and experimental animal breeding (AQUALABS, EL 91-BIO-03). Laboratory personnel include accredited technicians by the Federation for Laboratory Animal Science Associations (FELASA).

### Sample Collection

Sample collection information for the rudimentary sharpsnout seabream, and the protogynous common pandora and red porgy has been described in detail in Manousaki et al. (2014) and Tsakogiannis et al. (2018), respectively. The same procedure was followed for sample collection of the common dentex and the gilthead seabream. The fish used for the experiments were broodstock fish of wild origin in the Institute of Marine Biology, Biotechnology and Aquaculture (IMBBC, HCMR). The fish were euthanized using the commercially available slaughter method for marine fish (i.e., immersion in ice-slurry) and the brain and gonads were dissected immediately and kept in RNA-later (Qiagen) following provider's quidelines. The fish were examined macroscopically for sexual maturation, based on the presence of releasable sperm for males or the presence of vitellogenic oocytes for females and/or visual inspection of the gonad morphology. Specifically, for common dentex, brains and gonads from five mature females (average weight = 1.04 kg, age = 40 months old) and six mature males (average weight = 1.76 kg, age = 46 months old) were sampled (six biological replicates for the male gonads, five biological replicates for the male brains and five biological replicates for the females per tissue), while for gilthead seabream the same tissues were sampled from four mature males (average weight = 0.68 kg, age = 28 months old) and four mature females (average weight = 2.2 kg, age = 52 months old) (four biological replicates for each sex per tissue). Especially for common dentex, a pool of few hundred larvae at the “hatching” stage (1 day post-hatch, dph) was also sampled to enrich the species first transcriptome assembly (Table 1). In total, common dentex and gilthead searbeam sequences were obtained from 38 samples which were used for RNA extraction, library preparation, and sequencing.

**Table 1:** Samples and raw sequences of common dentex and gilthead seabream.

	**Biological replicates**	**Samples**	**Sex**	**Tissue**	**Raw reads**
Common dentex	5 female brains	DdFB1	Female	Brain	19,384,462
		DdFB2	Female	Brain	16,808,874
		DdFB3	Female	Brain	25,074,840
		DdFB4	Female	Brain	29,206,520
		DdFB5	Female	Brain	23,474,036
	5 female gonads	DdFG1	Female	Gonad	27,705,642
		DdFG2	Female	Gonad	24,365,178
		DdFG3	Female	Gonad	22,076,722
		DdFG4	Female	Gonad	21,219,416
		DdFG5	Female	Gonad	23,457,354
	5 male brains	DdMB1	Male	Brain	24,070,998
		DdMB2	Male	Brain	23,322,846
		DdMB4	Male	Brain	20,056,858
		DdMB5	Male	Brain	15,196,762
		DdMB6	Male	Brain	13,886,812
	6 male gonads	DdMG1	Male	Gonad	25,897,816
		DdMG2	Male	Gonad	34,311,430
		DdMG3	Male	Gonad	31,033,676
		DdMG4	Male	Gonad	25,901,108
		DdMG5	Male	Gonad	24,349,302
		DdMG6	Male	Gonad	21,256,004
	1 larvae pool	DdHatch	NA	Whole body	20,765,736
	Total	22	–	–	512,822,392
Gilthead seabream	4 female brains	SaFB1	Female	Brain	17,280,880
		SaFB2	Female	Brain	17,739,060
		SaFB3	Female	Brain	20,802,438
		SaFB4	Female	Brain	17,691,814
	4 female gonads	SaFG1	Female	Gonad	34,194,856
		SaFG2	Female	Gonad	33,241,228
		SaFG3	Female	Gonad	34,271,716
		SaFG4	Female	Gonad	32,293,436
	4 male brains	SaMB1	Male	Brain	20,938,852
		SaMB2	Male	Brain	28,548,174
		SaMB3	Male	Brain	25,808,438
		SaMB4	Male	Brain	28,432,110
	4 male gonads	SaMG1	Male	Gonad	33,073,506
		SaMG2	Male	Gonad	31,482,028
		SaMG3	Male	Gonad	32,654,702
		SaMG4	Male	Gonad	31,861,858
	Total	16			440,315,096

### RNA Extraction and Sequencing

Each one of the samples was ground with liquid nitrogen using pestle and mortar, homogenized in TRIzol® reagent (Invitrogen) and total RNA was extracted from the TRIzol® homogenate according to the manufacturer's instructions. RNA quantity was measured spectrophotometrically with NanoDrop® ND-1000 (Thermo Scientific), while its quality was tested first on agarose gel (electrophoresis in 1.5% w/v) and then on Agilent Technologies 2100 Bioanalyzer (Agilent Technologies). Finally, each one of the 38 RNA samples of common dentex and gilthead seabream was used for mRNA paired-end library construction with the Illumina TruSeq™ RNA Sample Preparation Kits v2 following manufacturer's guidelines. For common dentex, 22 cDNA libraries were sequenced in one and a half lane of an Illumina HiSeq2500™ instrument and run with a 100 bp paired–end manner. All 16 gilthead seabream libraries were sequenced on an Illumina HiSeq4000™ instrument with the 150 bp paired-end method in one lane following the protocols of Illumina Inc. (San Diego, CA). The raw sequences obtained for each sample are presented in Table 1.

### Bioinformatic Analysis

Raw reads were pre-processed following the pipeline published in Ilias et al. (2015). Initially, we used Scythe [https://github.com/vsbuffalo/scythe] to identify adapter contamination setting prior contamination rate set in 0.1 “-p 0.1,” Sickle to trim low quality reads (parameters “pe -g -t sanger -q 20 -l 45”), Trimmomatic (Bolger et al., 2014) (parameters “PE -phred33 ILLUMINACLIP:adapter_file.fa:2:30:10 LEADING:3 TRAILING:3 SLIDINGWINDOW:4:25 MINLEN:45 CROP:99”) and Prinseq (Schmieder and Edwards, 2011) to remove poly A/T tails and low complexity reads.

The two new assemblies were produced using Trinity v2.0.6 (Grabherr et al., 2011) with default parameters (default kmer 25, min length 200 nucleotides). Downstream assembly filtering was conducted using the same criteria as in Manousaki et al. (2014) (IsoPct < 1and FPKM < 0.3). To assess the quality of all five assemblies we used three criteria: (1) assessing the percentage of mapped reads of each species back to the assembly using Bowtie2 (Langmead and Salzberg, 2012) and Samtools (Li et al., 2009); (2) examining the similarity between the Trinity assembly and Swissprot database (UniProt Consortium, 2015) using blastx (Altschul et al., 1990) searches within the parallel NOBLAST program (Lagnel et al., 2009); and (3) quantifying the completeness of each assembly using BUSCO (search for “vertebrates” in OrthoDBv8) (Simão et al., 2015). The newly obtained assemblies were then functionally characterized as described in Tsakogiannis et al. (2018).

Differential expression (DE) analysis was performed at gene level by pairwise comparisons between male and female for brain and gonad samples in each species separately. The pre-processed reads of each species were aligned to the respective assembly with Bowtie2 (Langmead and Salzberg, 2012) and abundance was estimated with RSEM (Li and Dewey, 2011), as implemented in the Trinity script *align_and_estimate_abundance.pl*. The estimated expected counts for each sample, when the sum of counts in each row >15, were extracted (Bourgon et al., 2010). To accomplish the same consensus in differential expression (DE) analysis among all species, we re-analyzed sharpsnout seabream to bring into line with the other four species. Count data were imported in R (version 3.2.0) and the analysis of differential expression conducted in DESeq2 (version: 1.8.1) (Love et al., 2014), an R bioconductor package (Gentleman et al., 2004). Each species brain and gonadal samples were grouped according to sex and expression was compared (male vs. female), following the developers' manual (false discovery rate, FDR, threshold of 0.05). Genes with an adjusted *p*-value < 0.05 were considered significantly differentially expressed. Principal component analysis (PCA) (Jolliffe, 2002) and the heatmap.2 function in the gplots package (Warnes et al., 2009) were used to visualize global similarities and differences among the gonadal samples. Each species PCA plot was produced including all DE genes (Differentially Expressed genes) in the filtered (sum of counts in each row >15) count matrix.

Functional-enrichment analysis was performed to identify gene ontology (GO) terms and metabolic pathways significantly enriched in DE genes in gonads. Here, the term ‘enrichment’ stands for the comparison between the DE genes of the female (test-set) against the male (reference-set) and the gene-set was restricted to only those with |log_2_FC| >2. All enrichment analyses were implemented in BLAST2GO (Conesa et al., 2005) with default settings enabling a two-tailed test (FDR = 0.05).

To track down the expression patterns in known sex-related candidate genes in vertebrates and other fish taxa, we downloaded the respective genes of tilapia, medaka and spotted gar from Ensembl (release 84) (Yates et al., 2015) resulting in a set of 114 candidate genes (see also Tsakogiannis et al., 2018). To see if orthologs of these genes were present in our species transcriptomes, we implemented a reciprocal BLASTn best-hit approach (Overbeek et al., 1999). Potentially orthologous genes differentially expressed in all species or were species-specific were identified.

The final step included a comparative analysis for male and female-biased orthologous gonadal genes. Following an in-house built pipeline, using perl scripts and the Vennt tool (http://drpowell.github.io/vennt/) we combined the expression data of *D. dentex, S. aurata, P. pagrus, P. erythrinus, and D. puntazzo* gonadal orthologous genes.

We performed a BLASTn between each pair of the five transcriptomes (*e*-value threshold 10^−10^), keeping the best reciprocal hits (bidirectional best blast hits), an approach followed also in Tsakogiannis et al. (2018). We then selected all transcripts that have orthologs in all five species. Next, we combined the expression information with the existing orthologous genes. Following that, we visualized and analyzed the data using the freely available Vennt tool (version v0.8.4, GPL v.3, http://drpowell.github.io/vennt), which generates dynamic Venn diagrams for differential gene expression. Based on the Vennt results, we concluded that an FDR value lower than 0.05 produces robust results. Finally, we plotted a Venn diagram for all five species using the R package “venn” (version 1.5, GPL v.3, https://cran.r-project.org/web/packages/venn/index.html). A more detailed description of parameters used in bioinformatic analysis can be found in Supplementary Excel Table SET1.

## Results

### Pre-processing Treatment of Reads and Assembly Construction

Illumina sequencing of common dentex and gilthead seabream yielded 512,882,392 and 440,315,096 reads, respectively. The raw sequence data has been submitted to the National Center for Biotechnology Information (NCBI) Sequence Read Archive (SRA) database (common dentex: BioProject ID PRJNA481721 and gilthead seabream: BioProject ID PRJNA415944).

Reads' pre-processing led to 272,911,232 and 368,540,942 high-quality paired reads for common dentex and gilthead seabream, respectively. High-quality reads were used to construct the initial transcriptome assemblies, consisting of 247,693 transcripts for common dentex (N50: 1,891 nucleotides, mean length: 870 nucleotides) and 292,100 for gilthead seabream (N50: 1,939 nucleotides, mean length: 872 nucleotides).

**Figure 1:**
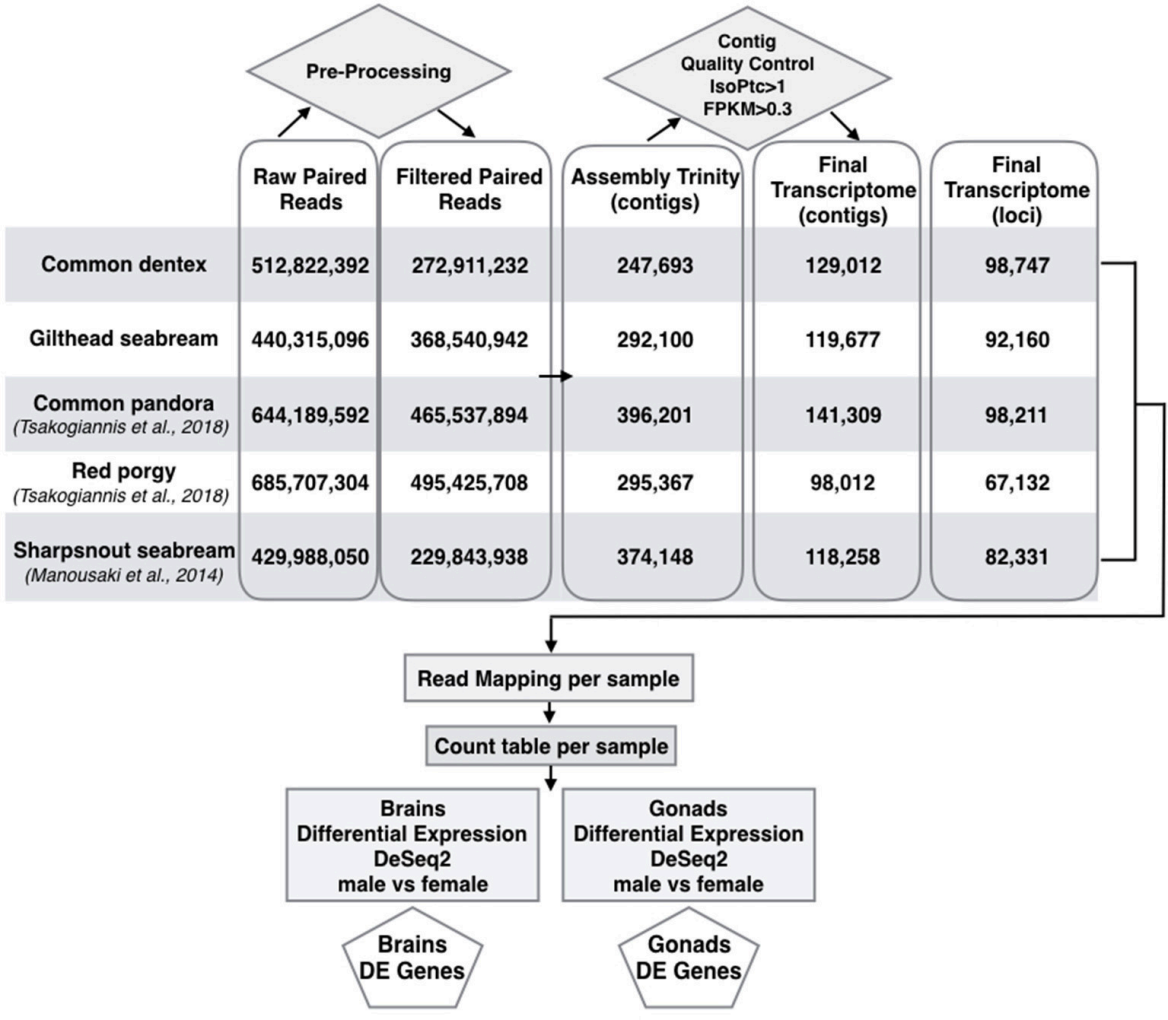
Pipeline for building assemblies and differential expression profiles in brains and gonads of the two species sequenced here in addition to sharpsnout seabream (Manousaki et al., 2014), common pandora and red porgy (Tsakogiannis et al., 2018). Flow chart of the basic steps implemented from raw reads to the selection of final assembled loci and DE genes in brains and gonads of all species.

Following transcript quality control, the initial assemblies were limited to 129,012 contigs (N50: 2,592; mean length: 1,259 nucleotides) corresponding to 98,747 genes for common dentex and 119,677 contigs (N50: 2,806; mean length: 1,321 nucleotides) corresponding to 92,160 genes for gilthead seabream. The restricted final datasets combined with the respective datasets of the previously published sparids are shown in a comparative representation in Figure 1.

To evaluate the accuracy of the assembled transcripts, clean reads were aligned against the transcripts, reaching relatively high percentages in both common dentex (85%) and gilthead seabream (80%). Then, a sequence similarity search between each species and UniProtKB/SwissProt database disclosed 19,429 and 16,690 contigs with hits for common dentex and gilthead seabream, respectively.

Finally, BUSCO run (Supplementary Table ST1) indicated that both assemblies were almost complete with 85% complete matches of vertebrate orthologs, found in both common dentex and gilthead seabream assemblies.

### Transcriptome Annotation

A blastx search of both assembled transcripts against UNIPROT (UniProt Consortium, 2015; *e*-value cut-off < 10^−5^) returned 26,509 and 24,326 genes with at least one hit for common dentex and gilthead seabream, respectively, and 24,248 and 23,961 genes with a protein domain match against InterProScan (Quevillon et al., 2005), respectively.

To identify possible functions, Gene Ontology (GO) assignments were used to classify the sequences. A total of 21,705 and 23,169 common dentex and gilthead seabream genes, respectively, were attributed with at least one GO term. The potential enzymes were characterized based on the chemical reaction they catalyze using the prediction of Enzyme Commission (EC) numbers for each sequence. In the case of common dentex 4,616 genes were classified to 1,037 different EC numbers, while in the case of gilthead seabream, 6,050 genes were classified to 1,215 different EC numbers (Table 2).

**Table 2:** Annotation summary of common dentex and gilthead seabream transcriptomes.

	**Annotation summary**							
	**Common dentex**				**Gilthead seabream**			
	**contigs**	**% contigs**	**genes**	**% genes**	**contigs**	**% contigs**	**genes**	**% genes**
	129,012		98,747		119,677		92,160	
BLASTx against Uniprot	46,471	36	26,509	27	39,049	33	24,326	26
InterPro	42,450	33	24,248	25	38,263	32	23,961	26
With IPR	38,378	30	21,986	22	34,251	29	21,462	23
With ≥ 1GO	30,720	24	17,717	18	27,437	23	17,102	19
Blast2Go annotated	34,595	27	21,705	22	37,535	31	23,169	25
EC	7,136	6	4,611	5	9,877	8	6,050	7

### Global Expression Pattern of Brains and Gonads

The global gene expression pattern observed for brain and gonad tissues for the common dentex and gilthead seabream is summarized by implementing a principal component analysis (PCA) (Supplementary Figure SF1). The plots show the formation of three separate clusters for each species, representing the brains, the male gonads and the female gonads. While male and female gonads show a clear separation with a remarkable variation within the two groups, male or female brains are clustered together exhibiting very similar expression patterns. The same pattern of samples clustering is also shown through heatmap plots (Supplementary Figure SF2).

### Sex-Biased Gene Expression in Male and Female Brains

**Figure 2:**
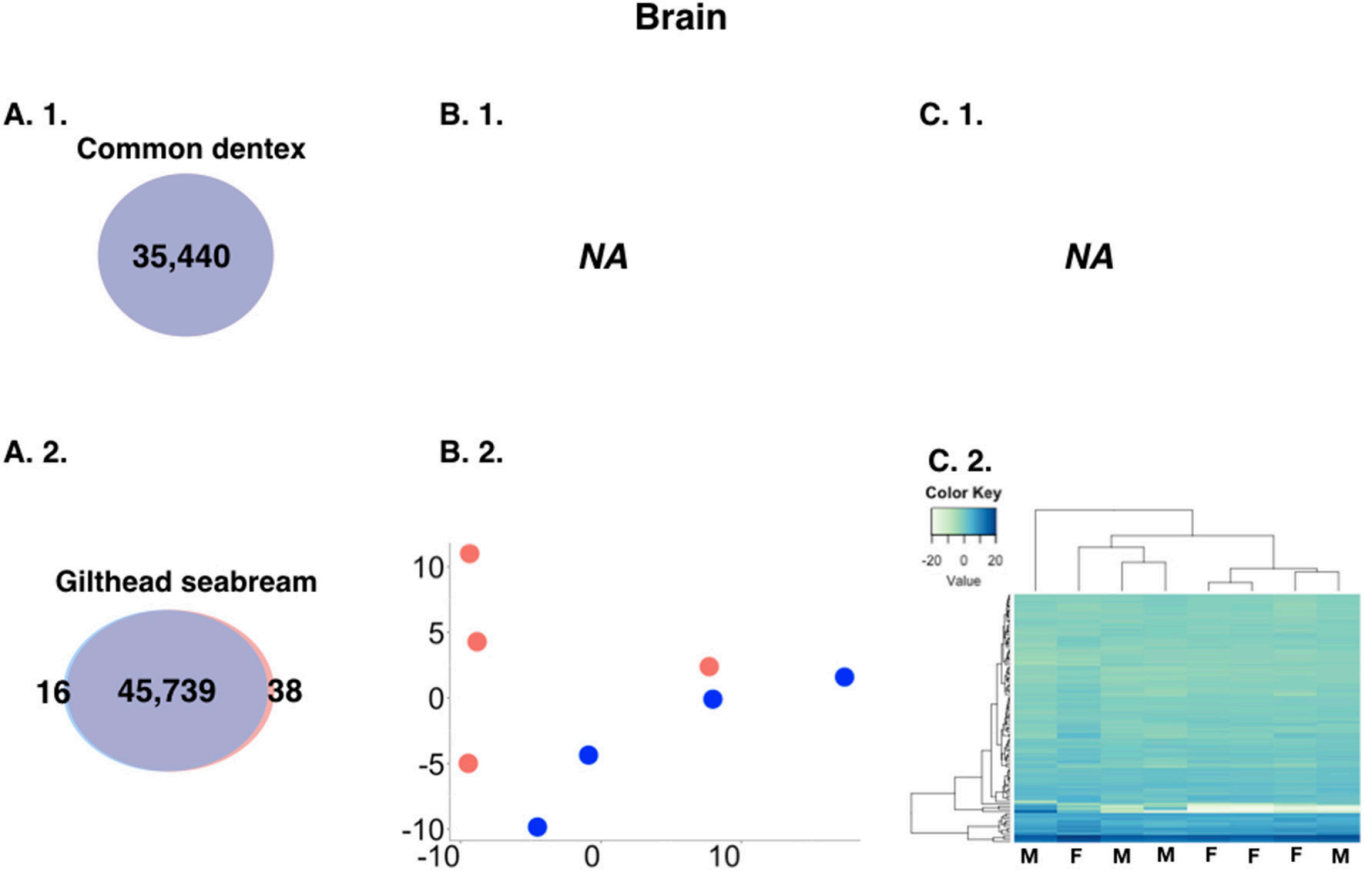
**(A)** Comparative view of the number of DE genes in brain between males and females in the two species. Venn-like diagram showing the DE genes in the two sexes in the brain of **(A. 1.)** common dentex and **(A. 2.)** gilthead seabream. Light-Blue: male-biased genes. Red: female-biased genes. Dark-Purple (intersection): un-biased genes. **(B)** Principal component analysis (PCA) plots in **(B. 1.)** common dentex and **(B. 2.)** gilthead seabream and **(C)** Heatmaps of the variance stabilized, transformed count data plots, for the DE genes in **(C. 1.)** common dentex and **(C. 2.)** gilthead seabream (M, Male; F, Female).

The analysis revealed minor differences in expression between sexes in the brain. For the purpose of the comparison, we kept only loci with a sum of expected counts in each row of the count matrix >15 leading to 35,440 and 45,793 brain genes for common dentex and gilthead seabream, respectively. In general, brain DE genes showed low counts and low fold-change differences. Common dentex showed no differential expression in the brain. In gilthead seabream, 54 genes were differentially expressed with 16 reported as male-biased and 38 as female-biased (Figure 2).

### Sex-Biased Gene Expression in Male and Female Gonads

**Figure 3:**
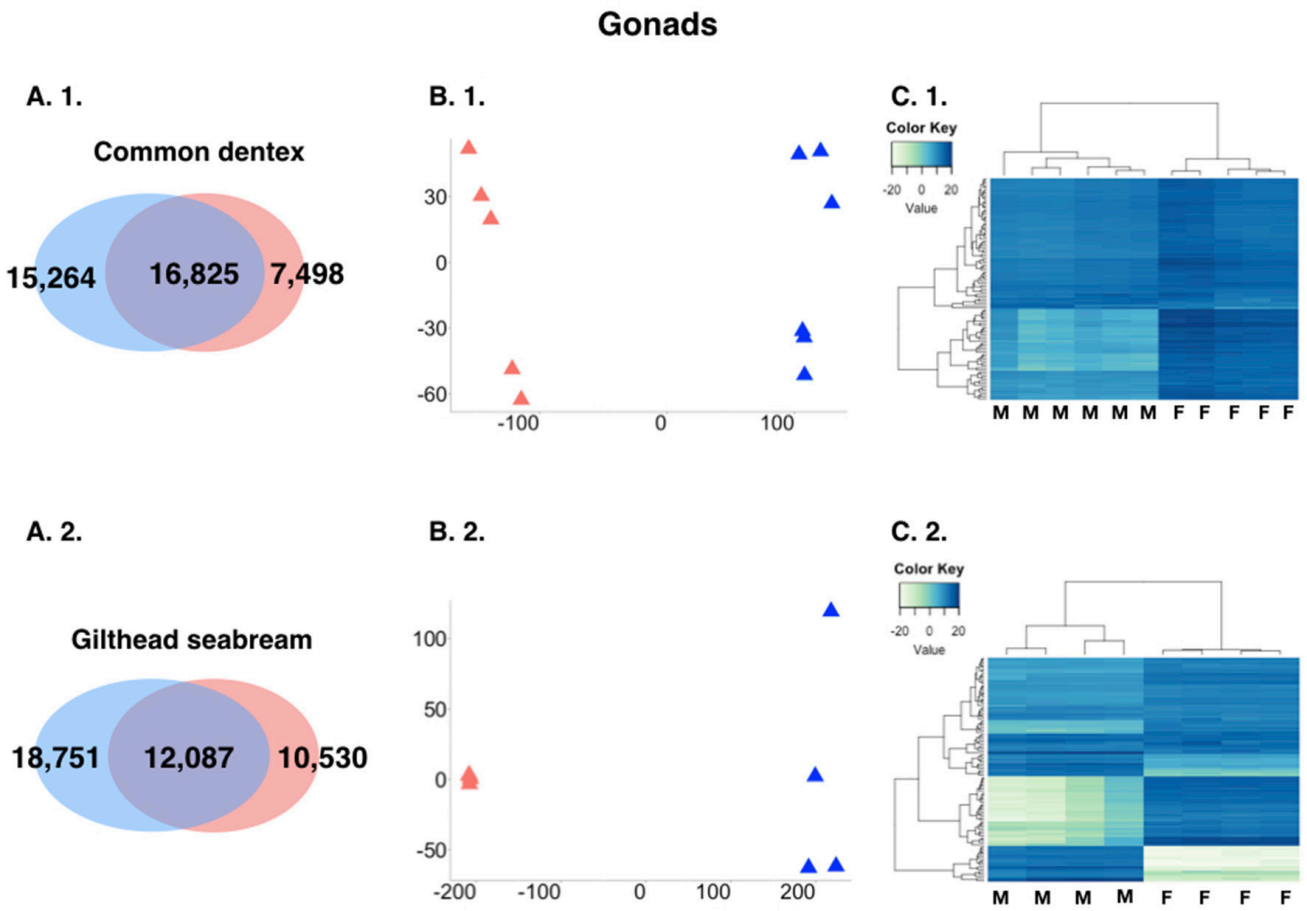
**(A)** Comparative view of the number of DE genes between males and females in the two species gonads. Venn-like diagram showing the DE genes in the two sexes in the gonads in **(A. 1.)** common dentex and **(A. 2.)** gilthead seabream. Light-Blue: male-biased genes. Red: female-biased genes. Dark-Purple (intersection): un-biased genes. **(B)** Principal component analysis (PCA) plots in **(B. 1.)** common dentex and **(B. 2.)** gilthead seabream and **(C)** Heatmaps of the variance stabilized, transformed count data plots, for the DE genes in **(C. 1.)** common dentex and **(C. 2.)** gilthead seabream (M, Male; F, Female).

The analysis revealed great differences in expression between male gonads and female gonads. For the purpose of the comparison, we kept only loci with a sum of expected counts in each row of the count matrix >15 keeping 39,587 and 41,368 for common dentex and gilthead seabream, respectively. More particularly, in the case of common dentex 22,762 genes were significantly differentially expressed with 15,264 over-expressed in males and 7,498 in females, while in the case of gilthead seabream 29,281 genes were differentially expressed with 18,751 over-expressed in males and 10,530 over-expressed in females (Figure 3).

For a better understanding of the biological processes underlying the nature of the functional male or female gonadal phenotype, GO term enrichment analysis was implemented. Enrichment Analysis of DE genes in common dentex revealed 230 GO terms significantly enriched in testis (light blue colored in Supplementary Excel Table SET2) and 316 GO in ovary (light red colored in Supplementary Excel Table SET2). In gilthead seabream, 366 GO terms were significantly enriched in testes (light blue colored in Supplementary Excel Table SET3) and 298 GO in ovaries (light red colored in Supplementary Excel Table SET3).

### Expression Profile of Reproduction-Related and Key- Candidate Genes in the Gonads

In order to identify genes that play an important role in reproduction we searched the annotated brain and gonad datasets for relevant GO categories and checked if they were differentially expressed. More particularly, DE genes related with broad biological processes terms, such as “*reproduction*” and “*reproductive process*” were searched in gonads of both sparids. A large number of “*reproduction*”-related and few “*reproductive process*”-related genes were found in the gonads of both species. In common dentex, 133 female-biased and 77 male-biased genes belonging to the GO term “*reproduction*,” and four female-biased and 14 male-biased genes belonging to “*reproductive process*” were detected. In gilthead seabream, 191 female-biased and 166 male-biased genes belonging to the GO term “*reproduction*,” and 19 female-biased and 20 male-biased genes belonging to “*reproductive process*” were detected (Supplementary Excel Tables SET4–12).

In an effort to identify the molecular machinery employed by each species to express the functional male or the female phenotype of the gonad, we aimed at analyzing and comparing the expression patterns of orthologous genes known for their involvement in the fish and the vertebrate sex determination/differentiation cascade. We selected 114 candidate genes in total. Combining data from the two newly sequenced species and the three sparids analyzed in Manousaki et al. (2014) and Tsakogiannis et al. (2018), we obtained a comparative view of expression of those genes in all five species (Table 3). Out of this list, 56 genes were present in the transcriptomes of all five species. Table 3 includes the 69 mostly discussed genes in other fish or vertebrates that were differentially expressed in at least one of the five species, and compares their expression pattern between them.

**Table 3:** Expression pattern of key- gonadal genes implicated in sex determination/differentiation of the five sparid species.

**Gene name**	**Gene description**	**Expression**					**Gene ID**				
		***P. pagrus***	***P. erythrinus***	***D. dentex***	***D. puntazzo***	***S. aurata***	***P. pagrus***	***P. erythrinus***	***D. dentex***	***D. puntazzo***	***S. aurata***
*Aldh1a2*	Aldehyde dehydrogenase 1 family, member A2	NSD	NSD	M	M	M	TR49490|c0_g1	TR70446|c0_g1	c52437_g1	comp94934_c0	TRINITY_DN57044_c0_g1
*Amh*	Anti-Müllerian hormone or Müllerian-inhibiting substance	NSD	M	(–)	M	NSD	TR66874|c1_g1	TR91164|c5_g4	NA	comp107049_c0	TRINITY_DN166272_c0_g1
*Amhr2*	Anti-Müllerian hormone receptor 2	F	NSD	M	M	M	TR66380|c1_g1	TR90446|c1_g4	c70823_g1	comp111277_c1	TRINITY_DN58797_c0_g1
*Ar*	Androgen receptor / Androgen receptor alpha	F	F	NSD	M	F	TR60985|c0_g1	TR83707|c1_g2	c72407_g2	comp108409_c1	TRINITY_DN41747_c0_g2
*Arb*	Androgen receptor beta	F	F	NSD	NSD	NSD	TR60273|c1_g1	TR85026|c0_g1	c73222_g3	comp122243_c0	TRINITY_DN60348_c4_g1
*Ctnnb1*	Catenin (cadherin-associated protein), beta 1	F	F	F	F	F	TR46279|c0_g1	TR64263|c0_g2	c56056_g2	comp112822_c5	TRINITY_DN28847_c0_g1
*Cyp11a2*	Cytochrome P450, family 11, subfamily A, polypeptide 2 / 11alpha-hydroxylase	F	NSD	NSD	F	M	TR61267|c0_g1	TR88875|c1_g5	c71096_g1	comp111704_c1	TRINITY_DN61832_c0_g2
*Cyp11b*	Cytochrome P450, family 11, subfamily C, polypeptide 1 / 11beta-hydroxylase	NSD	M	M	M	M	TR47689|c0_g1	TR72664|c0_g1	c64871_g2	comp108630_c0	TRINITY_DN6958_c0_g1
*Cyp19a1*	Aromatase a (gonad isoform)	F	F	F	F	F	TR50491|c0_g1	TR72019|c0_g1	c56116_g1	comp117077_c0	TRINITY_DN50138_c0_g2
*Cyp26a1*	Cytochrome P450, family 26, subfamily a, polypeptide 1	F	F	F	F	F	TR49995|c0_g1	TR72448|c0_g1	c62822_g1	comp104920_c0	TRINITY_DN35779_c0_g1
*Dax1*	Dosage-sensitive sex reversal, adrenal hypoplasia critical region, on chromosome X, gene 1	F	F	NSD	M	F	TR64306|c1_g1	TR19608|c0_g1	c55395_g1	comp87968_c1	TRINITY_DN25170_c0_g1
*Ddx11*	DEAD/H box helicase 11	M	M	NSD	NSD	NSD	TR66969|c0_g1	TR90413|c2_g1	c75063_g2	comp114371_c9	TRINITY_DN59935_c0_g1
*Ddx4*	Dead Box 4 Vasa Homolog	NSD	NSD	M	NSD	M	TR52887|c0_g1	TR69725|c0_g1	c59427_g1	comp116347_c5	TRINITY_DN54824_c0_g2
*Dhh*	Desert hedgehog	NSD	F	F	F	F	TR59771|c0_g1	TR84448|c0_g1	c62990_g1	comp116302_c0	TRINITY_DN25379_c0_g1
*Dmrt1*	Doublesex- and mab-3-related transcription factor 1	M	M	M	M	M	TR67635|c0_g1	TR70670|c0_g1	c71436_g2	comp110196_c1	TRINITY_DN51794_c0_g2
*Dmrt2a*	Doublesex- and mab-3-related transcription factor 2(a)	F	F	F	F	(–)	TR59371|c2_g1	TR78139|c1_g1	c64698_g2	comp112381_c0	NA
*Dmrt3a*	Double sex and mab-3 related transcription factor 3(a)	(–)	M	M	M	(–)	NA	TR67762|c1_g1	c65860_g1	comp88038_c1	NA
*Dnmt1*	DNA methyltransferase 1	F	F	F	F	F	TR62712|c0_g1	TR83768|c0_g1	c61611_g1	comp115925_c0	TRINITY_DN51749_c0_g1
*Dnmt3aa*	DNA methyltransferase 3aa	M	M	M	M	M	TR64267|c0_g1	TR73384|c7_g1	c70794_g1	comp120818_c3	TRINITY_DN58191_c0_g1
*Dnmt3ab*	DNA methyltransferase 3ab	F	F	M	M	NSD	TR53015|c0_g1	TR78244|c1_g1	c71723_g5	comp120102_c11	TRINITY_DN57426_c3_g1
*Ep300a*	Histone acetyltransferase—E1A binding protein 300a	M	M	NSD	NSD	M	TR60830|c1_g1	TR84051|c2_g6	c63663_g10	comp114282_c1	TRINITY_DN61531_c11_g1
*Ep300b*	Histone acetyltransferase—E1A binding protein 300b	NSD	NSD	NSD	(–)	F	TR62718|c3_g1	TR88233|c0_g14	c63663_g1	NA	TRINITY_DN61531_c7_g1
*Er*	Estrogen receptor	M	NSD	M	NSD	M	TR68522|c4_g2	TR84515|c0_g1	c71439_g2	comp117911_c10	TRINITY_DN43934_c0_g1
*Esr2a*	Type II estrogen receptor	NSD	NSD	M	NSD	F	TR65583|c1_g1	TR85716|c0_g1	c72247_g1	comp112065_c1	TRINITY_DN59537_c6_g2
*Esr2b*	Estrogen receptor 2b	(–)	(–)	M	(–)	(–)	NA	NA	NA	comp108758_c2	NA
*Esrra*	Estrogen-related receptor alpha	M	M	M	M	F	TR60672|c1_g2	TR86271|c1_g1	c68941_g2	comp112646_c1	TRINITY_DN60587_c0_g1
*Esrrb*	Estrogen-related receptor beta	F	F	F	NSD	F	TR66216|c1_g1	TR89871|c1_g3	c71889_g1	comp117190_c7	TRINITY_DN55451_c1_g1
*Esrrb2*	Estrogen receptor 2b	NSD	F	NSD	NSD	M	TR52662|c0_g1	TR77491|c0_g1	c61415_g2	comp108758_c0	TRINITY_DN53665_c0_g1
*Fgf20a*	Fibroblast growth factor 20a	NSD	NSD	NSD	M	(–)	TR56801|c3_g1	TR72320|c1_g1	c37432_g2	comp106110_c1	NA
*Fgf20b*	Fibroblast growth factor 20b	F	NSD	M	M	F	TR56801|c1_g1	TR73946|c0_g1	c62141_g1	comp85925_c1	TRINITY_DN108566_c0_g1
*Fgf9*	Fibroblast growth factor 9	M	NSD	NSD	(–)	(–)	TR66253|c2_g4	TR91211|c1_g5	c72708_g3	NA	NA
*Figla*	Factor in the germline alpha	F	F	F	F	F	TR49469|c0_g1	TR54099|c0_g1	c52499_g1	comp119797_c11	TRINITY_DN14620_c1_g1
*Foxl2*	Forkhead box L2	F	F	F	NSD	F	TR43506|c0_g1	TR59502|c0_g1	c59874_g1	comp95964_c0	TRINITY_DN52797_c0_g1
*Fsta*	Follistatin	F	F	M	M	(–)	TR48089|c0_g1	TR66770|c0_g3	c56990_g1	comp112642_c0	NA
*Fstl3*	Follistatin-like 3	NSD	NSD	M	M	M	TR64396|c0_g3	TR80964|c2_g1	c67341_g1	comp103373_c0	TRINITY_DN44642_c0_g1
*Fstl4*	Follistatin-like 4	NSD	NSD	F	M	NSD	TR66843|c3_g1	TR80451|c1_g1	c40110_g1	comp118767_c9	TRINITY_DN59298_c0_g1
*Fstl5*	Follistatin-like 5	M	M	M	NSD	NSD	TR60004|c0_g1	TR77519|c0_g5	c72310_g1	NA	TRINITY_DN64086_c1_g2
*FTZ-F1*	Steroidogenic factor-1/fushi tarazu factor-1	F	F	NSD	M	M	TR56092|c1_g1	TR80163|c1_g2	c67456_g3	comp120045_c3	TRINITY_DN50710_c0_g2
*Gata-4*	Gata-binding protein 4	NSD	F	NSD	M	M	TR63369|c1_g1	TR74516|c2_g1	c68365_g1	comp114090_c1	TRINITY_DN49474_c0_g2
*Gdf9*	Growth and differentiation factor 9	F	F	F	F	F	TR52139|c0_g1	TR70663|c0_g1	c59003_g1	comp69471_c0	TRINITY_DN8454_c0_g1
*Gsdf*	Gonadal soma derived factor	F	NSD	M	(–)	M	TR61049|c0_g1	TR72782|c0_g1	c67953_g1	NA	TRINITY_DN56147_c0_g1
*Hdac10*	Histone deacetylase 10	F	F	NSD	F	NSD	TR67490|c0_g1	TR88916|c0_g6	c74106_g2	comp104913_c0	TRINITY_DN55862_c0_g1
*Hdac11*	Histone deacetylase 11	F	NSD	M	M	F	TR66619|c2_g2	TR84940|c1_g2	c74380_g7	comp120490_c0	TRINITY_DN63334_c0_g1
*Hdac2*	Histone deacetylase 2	F	F	F	F	F	TR56322|c1_g2	TR66800|c1_g1	c70637_g5	comp104608_c0	TRINITY_DN47239_c0_g1
*Hdac7*	Histone deacetylase 7	F	F	NSD	M	F	TR57004|c0_g1	TR84823|c3_g1	c67700_g1	comp118197_c1	TRINITY_DN56857_c0_g1
*Hdac8*	Histone deacetylase 8	M	M	M	M	M	TR62362|c1_g1	TR86781|c2_g5	c67655_g1	comp111646_c0	TRINITY_DN59663_c6_g1
*Lhb*	Luteinizing hormone, beta polypeptide	F	F	F	F	F	TR40794|c1_g1	TR66625|c0_g1	c21134_g1	comp104837_c0	TRINITY_DN49327_c0_g1
*Lhr*	Luteinizing hormone receptor	F	F	NSD	M	F	TR61712|c0_g1	TR89660|c1_g2	c69306_g1	comp101199_c1	TRINITY_DN55453_c0_g1
*Nr3c1*	Glucocorticoid receptor	M	M	M	M	NSD	TR63170|c1_g1	TR84453|c3_g1	c69943_g1	comp110357_c2	TRINITY_DN30611_c0_g3
*Nr3c2*	Mineralocorticoid receptor	F	NSD	M	M	F	TR58538|c2_g1	TR84031|c1_g1	c70382_g1	comp109470_c2	TRINITY_DN45316_c0_g1
*Pdgfaa*	Platelet-derived growth factor alpha a	M	NSD	NSD	M	M	TR66514|c0_g1	TR87951|c1_g6	c73083_g1	comp113241_c2	TRINITY_DN57763_c0_g1
*Pdgfab*	Platelet-derived growth factor alpha b	F	F	NSD	M	(–)	TR64531|c0_g1	TR75590|c0_g1	c66673_g2	comp107178_c1	NA
*Pdgfba*	Platelet-derived growth factor beta a	F	F	NSD	M	NSD	TR58625|c0_g1	TR86429|c1_g1	c70504_g1	comp117159_c4	TRINITY_DN61779_c0_g1
*Pdgfbb*	Platelet-derived growth factor beta b	NSD	NSD	M	M	M	TR65607|c0_g1	TR87425|c0_g1	c67490_g1	comp113875_c0	TRINITY_DN40994_c0_g2
*Pdgfra*	Platelet-derived growth factor receptor, alpha	NSD	NSD	M	M	M	TR66258|c1_g1	TR88623|c1_g1	c71284_g1	comp116299_c0	TRINITY_DN59458_c0_g1
*Pdgfrb1*	Platelet-derived growth factor receptor, beta 1	NSD	NSD	M	M	M	TR64788|c0_g1	TR80831|c3_g2	c71970_g3	comp104719_c1	TRINITY_DN52984_c0_g1
*Pdgfrb2*	Platelet-derived growth factor receptor, beta 2	F	F	F	NSD	NSD	TR55622|c2_g1	TR83411|c3_g1	c73150_g1	comp113376_c0	TRINITY_DN57277_c0_g1
*Raraa*	Retinoid acid receptor alpha a	M	M	M	M	M	TR36645|c1_g1	TR82123|c4_g2	c64412_g3	comp113587_c1	TRINITY_DN36265_c0_g1
*Rarab*	Retinoid acid receptor alpha b	F	NSD	M	NSD	NSD	TR63371|c2_g1	TR84655|c1_g1	c68281_g8	comp120127_c2	TRINITY_DN45351_c0_g2
*Rarb*	Retinoid acid receptor beta	M	M	M	M	M	TR65946|c1_g2	TR81734|c1_g3	c72322_g2	comp119441_c0	TRINITY_DN63608_c1_g1
*Rspo1*	R-spondin-1	NSD	NSD	M	(–)	(–)	TR44782|c0_g2	TR43218|c0_g2	c50451_g1	NA	NA
*Sox3*	SRY-related HMG box 3	F	F	F	F	F	TR64334|c1_g1	TR69218|c2_g2	c60997_g2	comp87875_c1	TRINITY_DN61608_c6_g1
*Star-like*	Steroidogenic acute regulatory protein	NSD	F	M	M	NSD	TR53314|c0_g1	TR52301|c0_g1	c62942_g2	comp76502_c0	TRINITY_DN51715_c0_g2
*Stra6*	Stimulated by retinoic acid gene 6	F	F	F	F	F	TR59700|c3_g1	TR86270|c0_g1	c68491_g1	comp105129_c1	TRINITY_DN43771_c0_g1
*Tdrd1*	Tudor domain containing 1	NSD	M	M	M	M	TR68477|c1_g4	TR91962|c2_g5	c70984_g2	comp113472_c0	TRINITY_DN59161_c0_g1
*Tdrd7*	Tudor domain containing 7	M	M	M	M	M	TR63911|c0_g1	TR88488|c1_g2	c73678_g1	comp115842_c3	TRINITY_DN61324_c1_g1
*Wnt4a*	Wingless-type MMTV integration site family, member 4a	F	F	F	(–)	(–)	TR40636|c0_g1	TR67106|c0_g2	c15210_g2	NA	NA
*WT1*	Wilms tumor protein 1a	F	F	NSD	M	M	TR67279|c4_g5	TR89074|c0_g1	c64908_g1	comp117315_c1	TRINITY_DN59519_c2_g1
*WT1b*	Wilms tumor protein 1b	F	F	NSD	(–)	M	TR57899|c0_g1	TR89074|c1_g1	c67340_g1	NA	TRINITY_DN57126_c1_g1

*M, male-biased; F, female-biased; NSD, not significantly different*.

### Comparative Analysis of Sex-Biased Gene Expression in the Gonads of All Five Sparid Species

**Figure 4:**
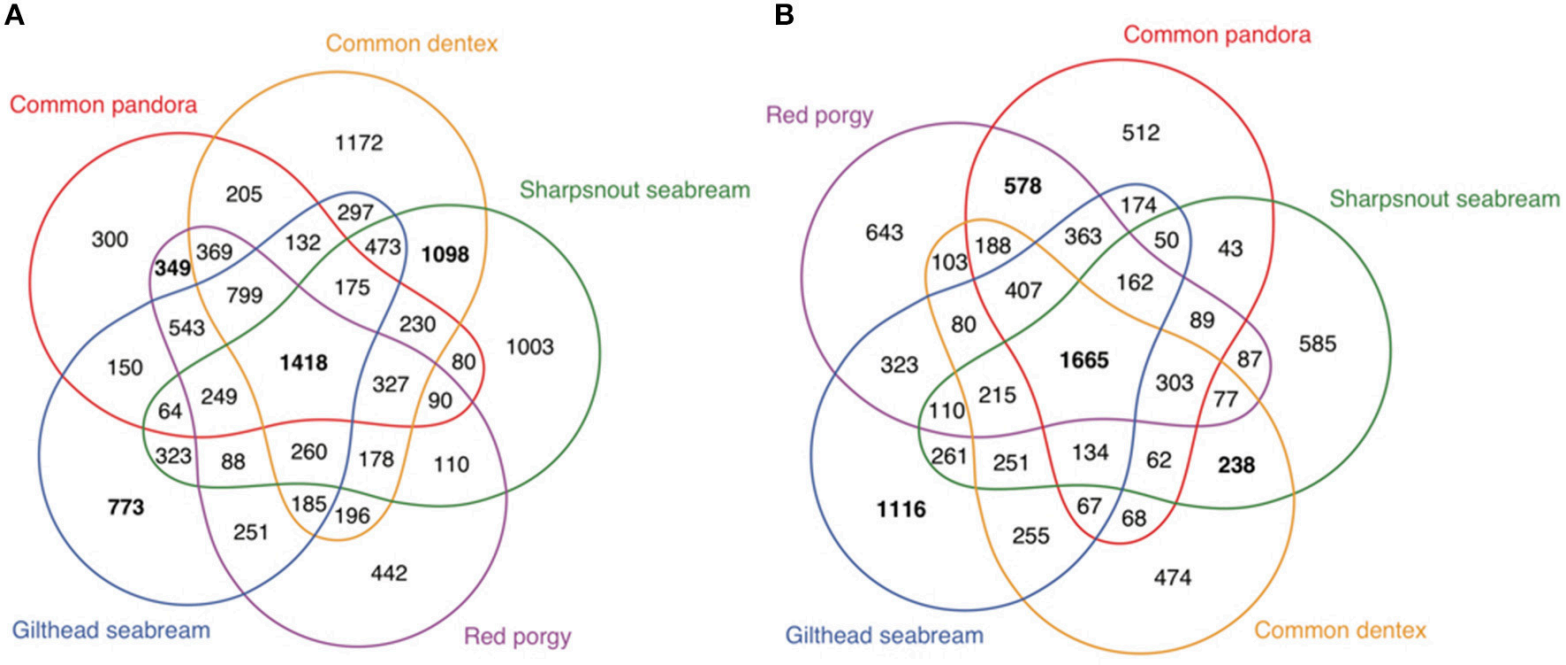
Multigroup comparisons of overexpressed genes in the gonads of all five sparids. The graphic shows the numbers of shared genes between species within the two groups of DE genes **(A)** testis overexpressed or male-biased and **(B)** ovary overexpressed or female-biased.

Apart from the 114 genes that were identified based on literature and public datasets, we looked for sex-biased differences using a comparative whole-transcriptome approach, based on the orthology assignment of the annotated transcriptomes of the gonads of all the species (Figure 4) (homologous genes between the five studied species in Supplementary Excel Table SET13). We compared the expression data of the annotated gonadal orthologous genes for the five species. Those multi-group comparisons helped us to identify common (in two, three, four, and all five species) and unique (species- specific) male- and female- biased genes in gonads. To gain greater insight into the genes that consistently play a role in gonadal identity we focused on genes that were differentially expressed in all five species (common/shared genes). The analysis revealed 1,418 annotated genes expressed in the testes (Figure 4A, intersection of all five species; Supplementary Excel Table SET14) and 1,665 annotated genes expressed in the ovaries (Figure 4B, intersection of all five species; Supplementary Excel Table SET15) of all five studied sparids. Moreover, this analysis allowed us to make comparisons of the testis- or ovary- specific genes between different reproductive modes. As such, one could focus on the protogynous-specific genes, with a shared over-expression in only the two protogynous species vs. all the other species. The analysis revealed 349 protogynous-specific orthologous genes overexpressed in testes (Supplementary Excel Table SET16) and 578 protogynous-specific orthologs overexpressed in ovaries (Supplementary Excel Table SET17). Considering that the sharpsnout seabream is a rudimentary or late gonochoristic species, the comparative analysis depicted 1,098 gonochoristic-specific male-biased orhologs (Supplementary Excel Table SET18), and 238 gonochoristic-specific female-biased orthologs (Supplementary Excel Table SET19). In an effort to gain a more profound insight into the protandrous-specific orthologs, the analysis produced 773 overexpressed genes in the testes (Supplementary Excel Table SET20) and 1,116 overexpressed genes in the ovaries of only the protandrous gilthead seabream (Supplementary Excel Table SET21). The maximum number of male-biased shared orthologs (1,098 genes) was found in the gonochoristic common dentex and rudimentary hermaphrodite sharpsnout seabream, when the maximum number of female-biased shared orthologs (578 genes) was found in the protogynous common pandora and red porgy.

## Discussion

The present study reports new transcriptome assemblies for two species of the Sparidae family, and compares the sex-specific gene-expression patterns in gonads and brains in four hermaphrodites and one gonochorist belonging to this family. This is the first comparative transcriptomic analysis of five phylogenetically close fish species displaying alternative reproductive modes. Focusing on genes that are expressed differentially between sexes, we pinpoint a common network that forms and/or maintains the functional male and female phenotype.

### Slight Differences Between Sexes in Brain. A “Sexually” Un-biased Tissue?

The analysis of the two newly sequence sparid species in this study confirms the insignificant sex-biased expression differences in the brain, reported previously in Manousaki et al. (2014) and Tsakogiannis et al. (2018). Generally, the DE genes in the brain are limited both in number and in expression magnitude. A limited number of DE genes between sexes were found in gilthead seabream brain, similar to those found in the rudimentary hermaphrodite sharpsnout seabream, whereas a total lack of sex-biased expression was observed in the gonochoristic common dentex. Conversely, the greatest differences in expression between males and females were observed in the brain of the two protogynous species. Regarding these protogynous species, it has been observed that the red porgy brain samples are more tightly clustered than those of common pandora that demonstrate a more relaxed clustering (Tsakogiannis et al., 2018). Moreover, the direction of expression was female-biased in the protogynous common pandora, the rudimentary hermaphrodite sharpsnout seabream and the protandrous gilthead seabream. These results are in line with the expression pattern observed in the protandrous clownfish *Amphiprion bicinctus* brain (Casas et al., 2016). In contrast, a male-biased pattern was observed here in the red porgy, as seen also in the protogynous bluehead wrasse *Thalassoma bifasciatum* (Liu et al., 2015) and four cichlid species (Böhne et al., 2014).

An interesting pattern arises when comparing the protogynous (highest sex-biased expression in brain), the protandrous and rudimentary hermaphrodite species (very low sex-biased expression in brain), and the gonochoristic species (no sex-biased expression in brain). However, the unbiased brain of the common dentex is not a common pattern in gonochoristic species, as this has not been previously reported in four gonochoristic East African cichlid species showing a low, but not zero sex-biased expression in their brain (Böhne et al., 2014). Generally, we found no consensus pattern among fish from the present and previous studies, indicating species-specific profiles in the brain. This has been previously observed and is linked to the great variation of sex-dependent behavioral and physiological traits in fishes (Maehiro et al., 2014).

A closer look at the specific genes that were up-regulated in male or female brains revealed numerous candidates with a well-known role in sex differentiation in other species. Some noteworthy genes that are putatively associated with male-like behavior were the vasotocin-neurophysin VT 1-like and isotocin-neurophysin IT 1-like, found among male-biased genes in the brain of gilthead seabream. We further searched in the trascriptomes of common dentex, gilthead seabream and sharpsnout seabream for the early growth response factor 1 (*Egr1*), the early growth response factor 4 (*Egr4*), as well as the proto-oncogene c-Fos (*Fos*), that were observed in the brain of the two protogynous species, the common pandora and the red porgy (Tsakogiannis et al., 2018). Interestingly, in gilthead seabream the *Fos* gene was female-biased and thus it might be indicative of the different behavior-related reproductive strategies between protandrous and protogynous species. These genes were also present in the brain transcriptomes of the rest of the species, but were unbiased. The *Egr1 and c-Fos* genes, both characterized as immediate-early genes, are related to changes in brain activity and genomic responses to social cues, behavior and mating information (Desjardins et al., 2010).

### Common Patterns of Gonadal Gene Expression in All Five Sparids

The highest degree of sex-biased gene expression is observed in the gonads (Mank et al., 2008). Despite the flexibility of gonadal tissue in sex-changers, the degree of difference between testes and ovaries is the same between the hermaphrodites and the gonochorist. Testes and ovaries are derived from the same sexually undifferentiated gonadal primordium, but once the sex has been established, even in hermaphrodites they represent two morphologically and functionally different organs. The transcriptomic profiles of the functional male and the female gonad differ in terms of the number of DE genes, as well as the expression magnitude (i.e., fold-change differences).

The male-biased expression tendency in gonads, applies in all studied sparids and it seems to be a common pattern in fish (Tao et al., 2013; Böhne et al., 2014; Manousaki et al., 2014; Liu et al., 2015; Casas et al., 2016; Machado et al., 2016; Xu et al., 2016; Tsakogiannis et al., 2018). Important signaling pathways such as WNT (wingless-type MMTV integration site family), NOTCH, TGFβ (transforming growth factor beta), nuclear receptors, for example the retinoic acid receptor (RAR), and RTKs (receptor tyrosine kinases) such as FGFR (fibroblast growth factor receptor), EGFR (epidermal growth factor receptor), and IR (insulin receptor) were all found to be among the significantly overrepresented terms of common dentex and gilthead seabream as well as in the rest of the species (Manousaki et al., 2014; Tsakogiannis et al., 2018).

The lists of enriched GOs were searched for biological processes related to the context of gonadal differentiation. Searching for terms related to hormone regulation we discovered several relevant terms over-represented in common dentex and gilthead seabream gonads such as: “*positive regulation of growth hormone receptor signaling pathway*,” “*positive regulation of hormone metabolic process*,” “*growth hormone secretion*” in testes of gilthead seabream and “*positive regulation of peptide hormone secretion*” in the testes of common dentex. Furthermore, androgen and estrogen related terms such as “*positive regulation of androgen secretion*,” “*androgen receptor signaling pathway*,” “*androgen metabolic process*,” “*estrogen biosynthetic process*” and “*response to estrogen*” were found to be enriched in the gonads of both common dentex and giltehead seabream. Among the female-specific GOs in both common dentex and gilthead seabream were the “*positive regulation of ovarian follicle development*,” while testis enriched terms in gilthead seabream were “*sperm motility*,” “*spermatid development*.” Interestingly, in common dentex female gonads the “*hermaphrodite genitalia development*” term was found.

Findings such as “response to retinoic acid,” “regulation of retinoic acid biosynthetic process,” “positive regulation of retinoic acid receptor signaling pathway” or “retinoic acid receptor signaling pathway” among both species gonads' enriched terms, indicate the role of the retinoic acid (RA) metabolism in gonadal development.

Furthermore, “*negative regulation of canonical Wnt signaling pathway*” and “*regulation of Wnt signaling pathway, calcium modulating pathway*” terms were enriched in ovaries of common dentex and gilthead seabream, respectively. Additionally, the term “*cellular response to transforming growth factor beta stimulus*” and “*positive regulation of transforming growth factor beta receptor signaling pathway*” was enriched in male gonads of common dentex and gilthead seabream, respectively. Known and described mostly in mammals (Windley and Wilhelm, 2015), these pathways apparently play an essential role in the context of sex differentiation and gonadal differentiation and development in the studied teleost species.

Lastly, in both common dentex and gilthead seabream, many epigenetic pathways are over-represented and seem to co-regulate reproductive processes. The enriched GO terms cover two major epigenetic mechanisms for gene expression regulation such as DNA methylation and histone modifications (acetylation, deacetylation).

### The Gonadal Transcriptomic Profile of Different Sex- Modes

**Figure 5:**
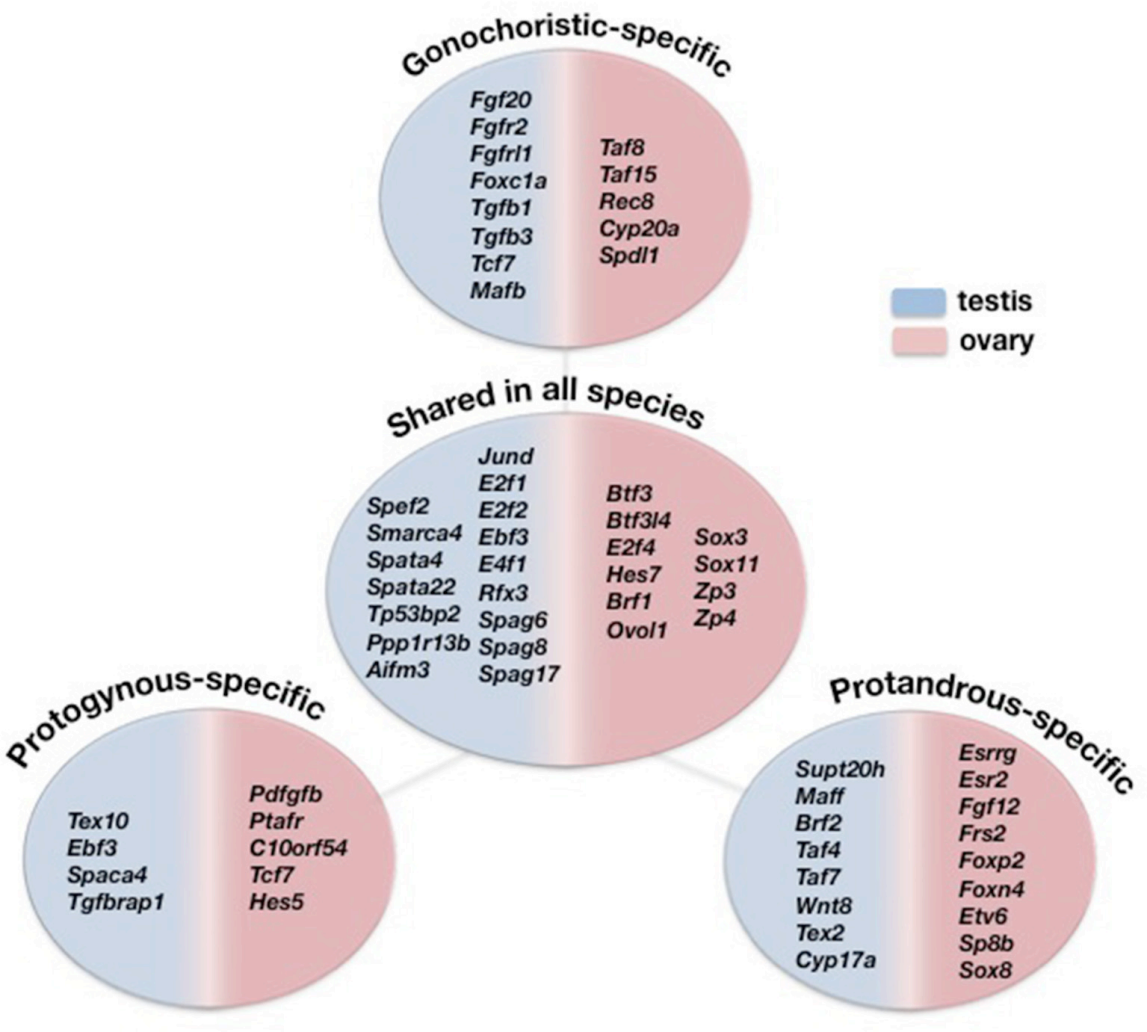
Comparative view of the DE genes overexpressed in testes (blue) and ovaries (pink) specific only to gonochoristic species (Common dentex and Sharpsnout seabream), to protogynous (Common pandora and Red porgy), to protandrous (Gilthead seabream) and shared among all five species.

A transcriptome-wide comparative analysis provided the opportunity to examine sex-biased expression profiles in five species displaying alternative reproductive modes and identify several interesting genes with sex mode-specific expression patterns (Figure 5). Focusing on the gonochoristic–specific (common dentex and sharsnout seabream) male-biased genes, several growth factor genes like *Fgf20, Fgfr2*, and *Fgfrl1* were found. Testis development requires the repression of *Wnt4* by *Fgf* signaling (Jameson et al., 2012). As we already know from mammals, *Fgf9* has an important function in male development, creating a positive feedback cycle with *Sox9*, inhibiting the WNT4 pathway in testis. The *Fgf9* gene has not yet been detected in teleosts and seems to be replaced by *Fgf20*, one of the genes involved in male sexual development also in *Latimeria* (Forconi et al., 2013). Among the gonochoristic-specific male-biased genes was also the *Foxc1a* that is required for primordial germ cell migration and antral follicle development in mice (Mattiske et al., 2006).

The analysis also revealed genes encoding for transcription factors such as *Tgfb1, Tgfb3, Tcf7*, and *Mafb*, where the former is considered to be expressed by the Sertoli cells and may be important for spermatogenesis (Memon et al., 2008), while, regarding the latter, studies in mice show that it is indispensable for the fetal testis morphogenesis and the maintenance of spermatogenesis (Shawki et al., 2018). The female-biased gonochoristic-specific genes included, among others, the TATA binding proteins *Taf8* and *Taf15*, the cytochoromic enzymes *Cyp20a and Spdl1* that is also a female-biased gene in the guppy, *Poecilia reticulata* (Sharma et al., 2014). Additionally, *Rec8* was found among the female-biased genes. This gene is present in all sequenced genomes of teleosts, including tilapia, but it is yet to be determined whether RA regulates germ cells meiotic initiation via *Rec8* in teleosts (Feng et al., 2015).

The protandrous-specific (gilthead seabream) male-biased genes included several transcription factors like *Supt20h, Maff*, *Brf2, Taf4, Taf7*. *Taf7* is one of genes shown to contribute to male fertility in mouse genetic studies and is expressed in spermatogenic cells (Cheng et al., 2007). A protandrous-specific gene expressed in testis were also the *Wnt8*, the testis expressed sequence 2, *Tex2*, and the *Cyp17a*. Recently, studies on mice detected *Cyp17* in germ cells: spermatogonia, pachytene spermatocytes, spermatids and sperm, and the loss of this enzyme is associated with sperm abnormalities and infertility. This suggests that, in addition to steroidogenesis, *Cyp17* may have a role in sperm structure and function (Hinfray et al., 2013).

On the other hand, the protandrous-specific female-biased genes included the estrogen receptors *Esrrg* and *Esr2*, the fibroblast growth factors *Fgf12* and *Frs2*, the forkhead box proteins *Foxp2* and *Foxn4* as well as the transcription factors *Etv6, Sp8b*, and *Sox8*. Interestingly, *Sox8*, a male determining factor, expressed uniquely in the ovaries of the protandrous gilthead seabream.

Among the protogynous-specific (common pandora and red porgy) male-biased genes were the testis expressed sequence 10, *Tex10*, the transcription factor *Ebf3*, the sperm associated protein *Spaca4* and the transforming growth factor b association protein *Tgfbrap1*. The protogynous–specific female-biased genes included the platelet-derived growth factor *Pdfgfb*, the platelet activating factor receptor *Ptafr* and the platelet receptor *C10orf54*. Of interest is that several transcription growth factors like *Tcf7, Hes5, Mafb* as well as *Sox7* were all among the female-biased genes in the two protogynous species while they were all male-biased in the gonochoristic common dentex and the late—gonochoristc species, sharpsnout seabream.

### Genes With a Conserved Role to Identify the Male and Female Character of the Gonad in Sparidae

The analysis provided us with more than a thousand distinct annotated orthologous genes in all five studied species, which might be regarded as potential candidates of gonadal sex identity in this group of teleosts and with a conserved role in identifying the male and female functional character of the gonad in Sparidae.

To gain more insight into the genes that have a conserved role in preserving the gonadal identity of the established sex at this group of teleosts we focused on a particular group, the genes that were DE in all five species (common/shared genes). Among the 1,418 annotated genes expressed in the testes, several genes coding for transcription factors like *Jund, E2f1, E2f2, Ebf3, E4f1*, and *Rfx3* and transcription activators like *Smarca4*, were found. There were also some testis associated proteins *Spag6, Spag8, Spag17* and spermatogenesis associated proteins like *Spata4* and *Spata22, Spef2* (the sperm flagellar protein 2), as well as some apoptotic factors like *Tp53bp2, Ppp1r13b*, and the *Aifm3* (apoptosis inducing factor). Among the 1,665 annotated genes expressed in the ovaries, there were the transcription factors *Btf3, Btf3l4, E2f4, Hes7, Brf1*, and *Ovol1* that are characterized by a specific and sustained high expression level in females compared to males, a finding that is also observed in the gonads of rainbow trout (Vizziano et al., 2007), including also the well-known *Sox* factors *Sox3* and *Sox11*. Additionally, the zona pellucida genes *Zp3* and *Zp4* were commonly over-expressed in ovaries of all five species.

Studies on other fish and vertebrates have identified several candidate genes with proven or presumed relationship in sex determination and differentiation. We compiled a list with some of the most- studied sex-biased genes and compared their expression patterns in the gonads of the studied sparids. The majority of the studied genes were present in the transcriptome of all five Sparidae species. Interestingly, there were some that showed a consensus bias in expression toward one sex. This might prove not only their association with the sex differentiation mechanism in fish but also their conserved roles in promoting either the female or the male character of the gonad in these species or, reductively, in this group of teleosts. The expression profiling brought to light well-known genes whose expression pattern jointly identifies the female (*Cyp19a1, Sox3, Figla, Gdf9, Cyp26a, Ctnnb1, Dnmt1, Stra6*) and male phenotype (*Dmrt1, Sox9, Dnmt3aa, Rarb, Raraa, Hdac8, Tdrd7*) of the gonad between these sparids.

Starting with the female phenotype of the gonad, that has long been assumed as the default state at least in mammals (Herpin and Schartl, 2011b), gonadal aromatase (*Cyp19a1a*) gene is a protagonist in the gene network controlling the gonadal fate. Here, a strong female-biased expression of gonadal aromatase gene (*Cyp19a1a*) was observed in all studied sparids. In fish, *Cyp19a1a* has a fundamental role in ovarian differentiation, both in gonochorists (Kitano et al., 1999; Uchida et al., 2004; Karube et al., 2007; Böhne et al., 2013), but also in hermaphrodites where its connection with sex-reversal is underlined (Kobayashi et al., 2010; Wu et al., 2010; Zhou and Gui, 2010; Shao et al., 2014; Baroiller and D'Cotta, 2016; Casas et al., 2016). The *Cyp19a1a* together with estrogens seem to control both ovarian and testicular differentiation in an adverse way. Over-expression of *Cyp19a1a* prompts and maintains female development, while its reduced expression allows for male development to occur (Guiguen et al., 2010). The *Cyp19a1a* that seems to act as a convergent read-out signifying commitment to the female pathway and responding to temperature in the European sea bass, represents a potential molecular link between the environment and the sex determination network (Navarro-Martín et al., 2011; Capel, 2017).

A factor promoting the formation and maintenance of the female phenotype, as it was commonly and strongly expressed in female gonads of all species of the present study, is that of *Figla (factor in germ-line alpha)*. The *Figla* gene encodes a transcription factor that can coordinate the expression of the oocyte-specific zona pellucida genes and folliculogenesis (Liang et al., 1997). Besides its well-accepted role in promoting ovarian development, *Figla* has also the ability to repress spermatogenesis and, thus acts as an antagonistic factor of male genetic hierarchies (Qiu et al., 2015). In hermaphrodites, its anti-parallel direction of expression to *Dmrt1* during the transition from male to female in a protandrous (Wu et al., 2012) and *vice versa* in a protogynous teleost (Miyake et al., 2012) has been suggested.

Likewise, the Luteinizing hormone (*Lh*) showed a strong female over-expression in all species examined here. Our findings, combined with studies in medaka, that have showed that *Lh* gene is involved in fish ovulation (Ogiwara et al., 2013), indicate that this gene has a conserved role to foster ovulation in wide range of fish even in phylogenetically remote species.

Another female-specific transcription factor is the growth differentiation factor-9 (*Gdf9*). *Gdf9* displayed sex-biased expression and was up-regulated in the ovaries of all studied species. This member of (TGFβ) superfamily, is an oocyte-specific factor in different vertebrates including fish (Elvin et al., 1999; Johnson et al., 2005; Liu and Ge, 2007). In mice, it is required in ovarian folicullogenesis (Dong et al., 1996), while in zebrafish it might also act as female-state protector by suppressing apoptosis; a mechanism responsible for degeneration of the ovarian tissue to pave the way to testicular development (Chen et al., 2017).

The common female “armory” is also assisted by a basic component of the *Wnt/b-catenin* pathway, the catenin beta 1 (*Ctnnb1*) gene. The present study confirms that the *Wnt/b-catenin* pathway has an active role in gonadal differentiation in all studied species (enrichment analysis). *Ctnnb1* showed a consistent pattern with a clear female-biased expression in these sparids thus proposing a conserved and acute role of *Ctnnb1* in preserving gonad's female identity in this fish lineage.

Similarly, the role of retinoic acid (RA) signaling pathway in gonadal differentiation has been well-stated (Yamaguchi and Kitano, 2012; Rodríguez-Marí et al., 2013; Feng et al., 2015). The implication of this pathway in fish sex determination remains largely unknown, and data from different species show that the enzymes responsible for RA catabolism may vary (Feng et al., 2015). In the current study, two pathway members, *Cyp26a* and *Stra6* showed a female-biased expression in all studied sparids. On the other hand, a search for genes encoding RA receptors revealed two retinoic acid receptors (*Raraa* and *Rarb*) that were male-biased in all studied sparids.

Evidence suggests that both Wnt/b-catenin and retinoic acid pathways are implicated in the sex differentiation process in the studied species. In the present study it is shown that these pathways are not exclusively female-specific but rather “modules” of each of them could be used to assist the development of both sexes. Indeed, the female specificity of the Wnt/b-catenin pathway in fish, as is also known in mammals (Chassot et al., 2014), is not a standard (Nicol et al., 2012).

Two recognized members of the *So*x gene family, *Sox3* and *Sox9*, were found among the commonly expressed gonadal genes in all the studied species. The present study demonstrates that *Sox3* had a high female-specific expression in the gonadal transcriptomes of all five sparids. *Sox* genes are highly conserved across evolution and encode a group of proteins that are well-known transcriptional regulators, involved in the decision of cell fates during development and in the control of diverse developmental processes (Pevny and Lovell-Badge, 1997). The *Sox3* ability to act as a trigger and its suitability for generating new sex-determining genes (Marshall Graves and Peichel, 2010) renders it a unique candidate gene that could be used as a master regulator but also as a more downstream factor in the SD cascade. The exact function of *Sox3* in gonadal differentiation in this group of teleosts remains to be elucidated, whereas in the newly analyzed species, common dentex and gilthead seabream, it is verified that it clearly supports female development.

The other Sox gene, Sox9, was found among the commonly expressed male-biased gene in all five fish studied here. Information from two other hermaphroditic fish, the protogynous bluehead wrasse (Liu et al., 2015) and the protandrous clownfish (Casas et al., 2016), support *Sox9* male-specific expression. In contrast, the medaka *Sox9* is expressed in ovarian oocytes (Yokoi et al., 2002) whereas in zebrafish this gene has two homologs, one expressed in oocytes of the ovary and the other in testicular Sertoli cells (Chiang et al., 2001). Here, the medaka *Sox9* ortholog, while found in the gonadal transcriptomes of all sparids, did not show any sex-biased expression. Interestingly, the tilapia *Oreochromis niloticus* and the *Tetraodon nigroviridis* orthologs of *Sox9a* were unanimously male-biased. This gene in mammalian systems is involved in testis differentiation, as well as in male fertility maintenance (Jiang et al., 2013). The redundancy that exists among *Sox* transcription factors with different *Sox* members activating similar target genes (Windley and Wilhelm, 2015) and also the expansion of this gene family in teleost fishes after the teleost-specific WGD (Voldoire et al., 2017), render them convenient molecular tools. Thus, it is possible that slightly different genes or gene copies, following a lineage- or even species-specific functional divergence, could play the role that *Sox9* plays in mammals. Taken together, the results suggest that *Sox3* and *Sox9* have a conserved role in the formation of the functional female and the male phenotypes of the gonad, respectively, in Sparidae, but also suggest that both genes are “convenient” molecular players that could contribute to either sex side in teleosts.

If the sex determination/differentiation process could be viewed as a struggle for prevalence between the male and female hierarchies (Herpin and Schartl, 2011b), then *Dmrt1* is an evolutionary conserved “molecular soldier” that stands in the battlefront, fighting for the male side in both vertebrates and invertebrates (Raymond et al., 2000; Marshall Graves and Peichel, 2010; Herpin and Schartl, 2011a; Matson and Zarkower, 2012). In the present study, *Dmrt1* was a highly over-expressed gene in testes of all studied sparids. In accordance with our results, its characteristic male-biased expression has been reported in numerous other gonochorists (Marchand et al., 2000; Fernandino et al., 2008; Ijiri et al., 2008; Berbejillo et al., 2012) as well as hermaphrodite teleosts (He et al., 2003; Zhou and Gui, 2010; Liu et al., 2015; Casas et al., 2016). Thus, the *Dmrt1* role in the regulation of male development seems to be conserved not only in gonochorism but also in hermaphroditism. Characterized by a conserved DNA-binding motif [Doublesex- and Mab- 3-related (DM) domain] and acting as a transcription factor, *Dmrt1* over-expression not only endorses male development by activating male-promoting genes, but also maintains male fates by repressing multiple female-promoting genes (Matson et al., 2011). An important function of *Dmrt1* is to actively and continuously suppress female gonad fates, thus maintaining the male fate (Herpin and Schartl, 2011b; Matson et al., 2011). The study of Shao et al. (2014) (Shao et al., 2014) implicates *Dmrt1* with methylation-related epigenetic modifications and suggests that is a critical gene that responds to environmental change and triggers the sex reversal cascade in tongue sole. Such action designates *Dmrt1* as a key player in “flexible” reproductive mechanisms like those in the studied sparid fishes for which sex is naturally reversible. Apparently, *Dmrt1* together with *Sox9* seems to signify the male character of the gonad in this fish lineage. It is now evident that the environment can affect the chromatin structure and DNA accessibility without causing actual nucleotide changes (Jaenisch and Bird, 2003). Genes encoding for epigenetic factors were abundant in the transcriptomes of all sparids. Epigenetic maintainers, such as the DNA-modifying enzymes DNA methyltransferases (*Dnmt3aa, Dnmt1, Dnmt3ab*) and histone tail-modifying and de-modifying enzymes, such as histone acetyl transferases (*Ep300a, Kat2b*) or histone deacetylases (*Hdac2, Hdac8, Hdac10, Hdac11*) were found in the gonads and were sex-biased. We are still far from understanding exactly how the epigenetic marks work, but since expression of most of the afore-mentioned genes is regulated from such factors, there is a strong indication that they play an integral role in shaping and/or maintaining the gonadal phenotype.

## Conclusions

In this study, we reported the transcriptome of gonads and brains of both sexes in two fishes of the Sparidae family, and the results were studied jointly with published transcriptomes of three other sparid species showing different reproduction modes (gonochoristic and protogynous hermaphrodites). This approach permitted us to obtain a global view of the transcriptome and to compare sex-specific expression patterns, as well as sex-biased gene expression patterns within fishes having alternative reproductive modes. The minor sex-related expression differences in the brain, when studied as a whole, might indicate a sexually un-biased tissue. On the contrary, in the gonads there was a plethora of differentially expressed genes between the sexes. This indicates functional divergence of the ovary and testis, even though both gonads originated from the same precursor tissue and despite the plasticity of the gonadal tissue in sex-changing species. Focusing on pathways and genes, we attempted to unveil the molecular means through which these fish species develop into males or females, and observed the recruitment of known sex-related genes either to male or female type of gonads in these fish. Taken together, most of the studied genes form part of the cascade of sex differentiation and reproduction across teleosts. The knowledge obtained in this study and tools developed during the process have laid the groundwork for future experiments that can improve the sex control of this species and help the in-depth understanding of the complex process of sex differentiation in the more flexible multi-component systems such as these studied here.

## Availability of Supporting Data

The datasets generated and/or analyzed during the current study are available from the corresponding author on request. The raw sequence data has been submitted to the National Center for Biotechnology Information (NCBI) Sequence Read Archive (SRA) database (common dentex: BioProject ID PRJNA481721, and gilthead seabream: BioProject ID PRJNA415944). The transcriptomic sequences can be retrieved under the following accession numbers: SRR7536480- SRR7536499, SRR7536500- SRR7536502 for common dentex and SRR6223527-SRR6223532, SRR6223535-SRR6223542 for gilthead seabream.

## Author Contributions

AT, TM, CM, and CT designed experiments. AT, CT, NPS, and CM conducted sampling. AT performed laboratory experiments. AT, TM, NPu, and JL analyzed data. AT wrote the manuscript. CT conceived and managed the project. All authors read and approved the final version of the manuscript.

### Conflict of Interest Statement

The authors declare that the research was conducted in the absence of any commercial or financial relationships that could be construed as a potential conflict of interest.
