# Supplementary figures and images for: The Gene Toolkit Implicated in Functional Sex in Sparidae Hermaphrodites: Inferences From Comparative Transcriptomics
Source: Front Genet. 2019 Jan 18;9:749. doi: 10.3389/fgene.2018.00749 (PMC6345689; doi:10.3389/fgene.2018.00749)

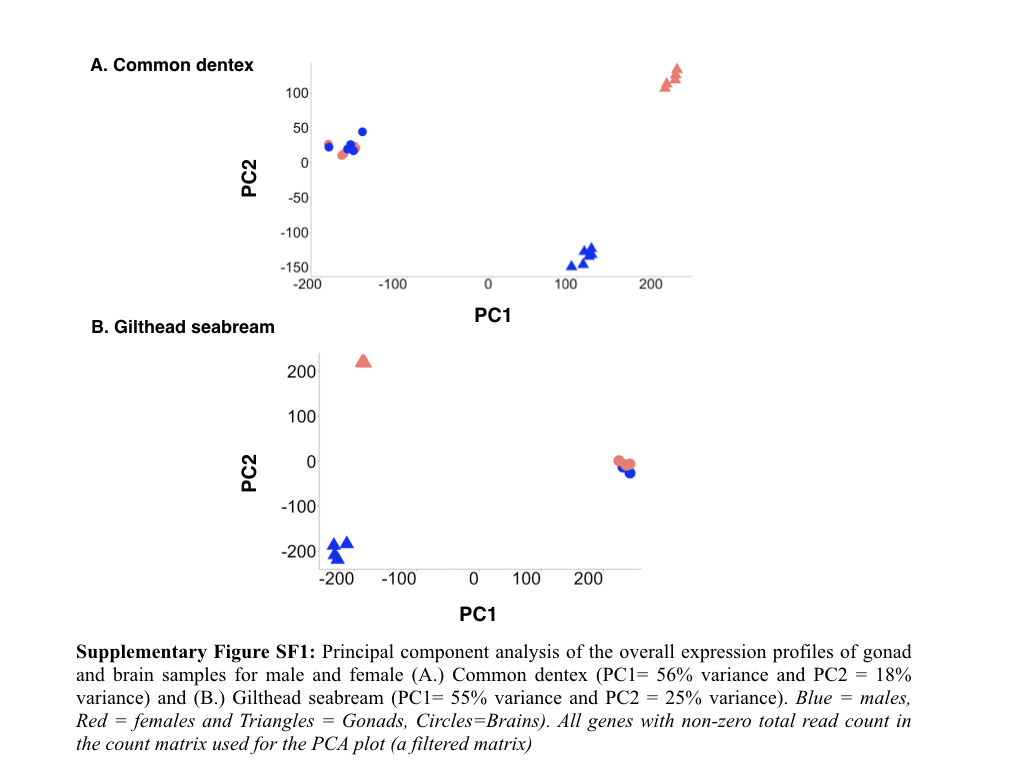

Supplement: Supplementary file 5 [file Image_1.JPEG]

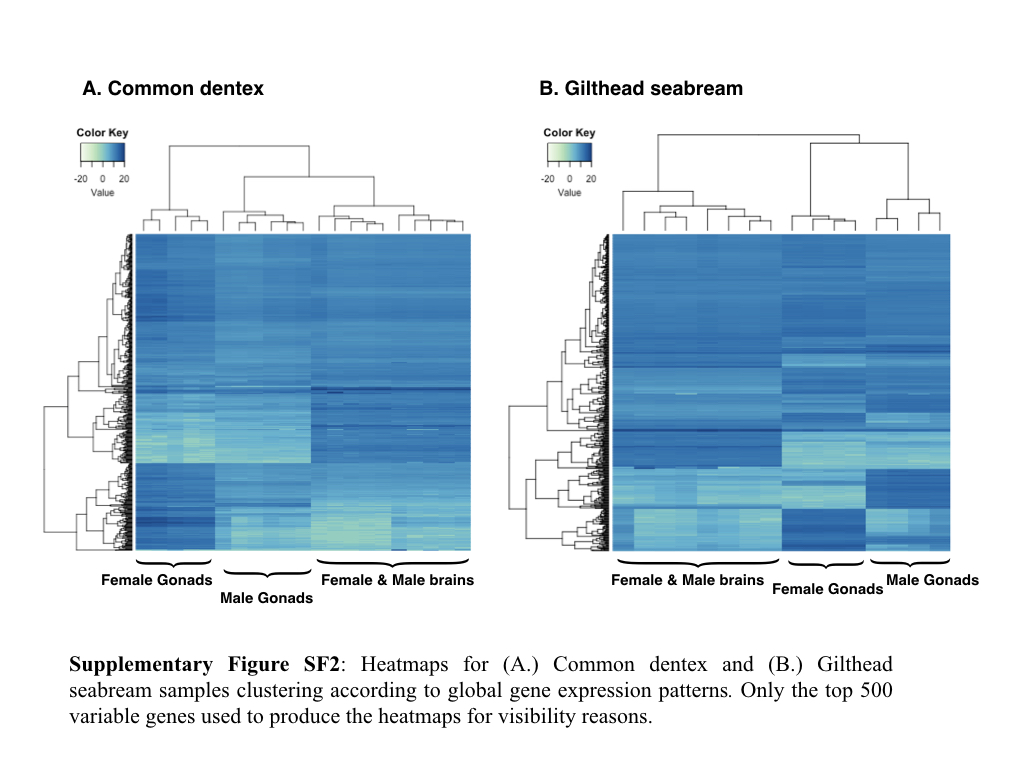

Supplement: Supplementary file 6 [file Image_2.JPEG]

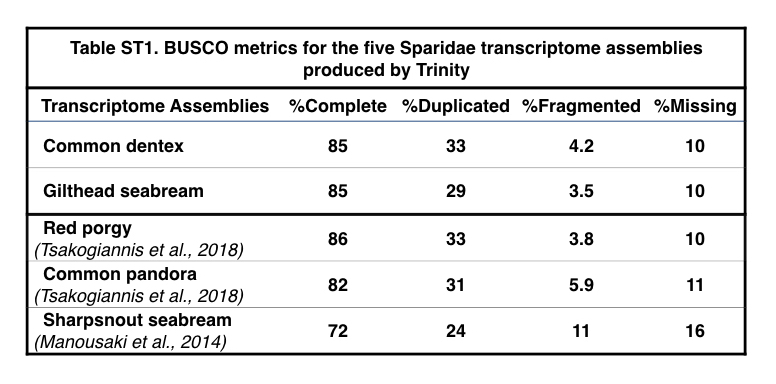

Supplement: Supplementary file 7 [file Image_3.JPEG]
